# Pharmacological analysis of the increases in heart rate and diastolic blood pressure produced by (*S*)-isometheptene and (*R*)-isometheptene in pithed rats

**DOI:** 10.1186/s10194-017-0761-y

**Published:** 2017-05-04

**Authors:** Alejandro Labastida-Ramírez, Eloísa Rubio-Beltrán, Oswaldo Hernández-Abreu, Bruce L. Daugherty, Antoinette MaassenVanDenBrink, Carlos M. Villalón

**Affiliations:** 1 Department of Pharmacobiology, Cinvestav-Coapa, Czda. Tenorios 235, Col. Granjas-Coapa, Deleg. Tlalpan, 14330 Mexico City, Mexico; 2 Tonix Pharmaceuticals, Inc. 509 Madison Avenue, Suite 306, New York, NY 10022 USA; 3 Division of Vascular Medicine and Pharmacology, Erasmus University Medical Center, P.O. Box 2040, 3000 CA Rotterdam, The Netherlands

**Keywords:** Isometheptene, Enantiomers, Vasopressor, Adrenoceptors, Rats

## Abstract

**Background:**

Isometheptene is a sympathomimetic drug effective in acute migraine treatment. It is composed of two enantiomers with diverse pharmacological properties. This study investigated in pithed rats the cardiovascular effects of (*S*)- isometheptene and (*R*)-isometheptene, and the pharmacological profile of the more potent enantiomer.

**Methods:**

The effects of i.v. bolus injections (0.03, 0.1, 0.3, 1 and 3 mg/kg) of isometheptene racemate, (*S*)-isometheptene or (*R*)-isometheptene on heart rate and blood pressure were analyzed in control experiments. The enantiomer producing more pronounced tachycardic and/or vasopressor responses was further analyzed in rats receiving i.v. injections of prazosin (0.1 mg/kg), rauwolscine (0.3 mg/kg), propranolol (1 mg/kg) or intraperitoneal reserpine (5 mg/kg, -24 h).

**Results:**

Compared to (*R*)-isometheptene, (*S*)-isometheptene produced greater vasopressor responses, whilst both compounds equipotently increased heart rate. The tachycardic responses to (*S*)-isometheptene were abolished after propranolol, but remained unaffected by the other antagonists. In contrast, the vasopressor responses to (*S*)-isometheptene were practically abolished after prazosin. Interestingly, after reserpine, the tachycardic responses to (*S*)-isometheptene were abolished, whereas its vasopressor responses were attenuated and subsequently abolished by prazosin.

**Conclusions:**

The different cardiovascular effects of the isometheptene enantiomers are probably due to differences in their mechanism of action, namely: (i) a mixed sympathomimetic action for (*S*)-isometheptene (a tyramine-like action and a direct stimulation of α_1_-adrenoceptors); and (ii) exclusively a tyramine like action for (*R*)-isometheptene. Thus, (*R*)-isometheptene may represent a superior therapeutic benefit as an antimigraine agent.

## Background

Isometheptene racemate is a sympathomimetic amine that has been used for decades in the acute treatment of moderate primary headaches (i.e. migraine and tension-type headaches) [1, 2], but its exact mechanisms of action have not been comprehensively explored. In this respect, our group has demonstrated in pithed rats [3] and anesthetized dogs [4] that isometheptene racemate behaves as a dual-acting sympathomimetic agent producing: (i) an indirect vasoconstrictor tyramine-like action (attenuated by reserpine) that involves the displacement of endogenous noradrenaline from sympathetic nerves; and (ii) a direct vasoconstriction that results in either rat vasopressor responses mediated by α_1_-adrenoceptors [3] or decreases in canine external carotid blood flow/conductance mediated by α_2A/2C_-adrenoceptors [4]. These properties have linked isometheptene’s therapeutic effectiveness with the vascular theory of migraine [5], so that its cranial vasoconstriction may reduce the phenomenon (i.e. vasodilatation) that has been advocated as a mechanism of headache and associated symptoms [6]. Interestingly, sumatriptan (a 5-HT_1B/1D_ receptor agonist with acute antimigraine properties) seems to exert part of its antinociceptive action by constricting the human middle meningeal artery (MMA) [7, 8]. In contrast, isometheptene racemate failed to constrict the porcine MMA [9], but the fact that it produces severe arterial vasospasm [10, 11] supports its vasoactive action on dilated cranial and cerebral arterioles [12, 13].

Tonix Pharmaceuticals has separated isometheptene racemate into its two enantiomers (*R*)-isometheptene and (*S*)-isometheptene, in order to explore their efficacy in the acute treatment of primary headaches [14]. Isometheptene racemate binds with high affinity (K_i_ = 42 nM) to the I_1_-imidazoline receptor (I_1-_R), suggesting that this receptor, along with α-adrenoceptors, could be involved in its antimigraine action [15]. Moreover, (*R*)-isometheptene binds to the I_1_-R (K_i_ = 18 nM) with about 60-fold greater affinity than (*S*)-isometheptene (K_i_ = 1100 nM) [15]. Notably, other studies have described: (i) a decreased pain threshold in I_1_-R knockout mice [16]; and (ii) that treatment with (*R*)-isometheptene relieved trigeminal sensitivity in the inflammatory soup and in spontaneous trigeminal allodynia rat models, two different models of chronic migraine [17]. This study investigated the cardiovascular effects produced by these novel compounds in an experimental model predictive of systemic (cardio) vascular side effects.

## Methods

### Animals

Seventy-four male normotensive Wistar rats (250–300 g, 8–10 weeks of age) were maintained at a 12/12-h light–dark cycle (with light beginning at 07:00 h) and housed in a special room at a constant temperature (22 ± 2 °C) and humidity (50%), with food and water ad libitum in their cages. All animal protocols of this investigation were approved by our Institutional Ethics Committee (CICUAL-Cinvestav; permission protocol number 507–12) and followed the regulations established by the Mexican Official Norm (NOM-062-ZOO-1999), in accordance with ARRIVE (Animal Research: Reporting In Vivo Experiments) reporting guidelines for the care and use of laboratory animals.

### General methods

After anaesthesia with sodium pentobarbital (60 mg/kg, i.p.) and cannulation of the trachea, all rats were pithed by inserting a stainless steel rod through the orbit and foramen magnum and down the vertebral foramen, as previously reported [18, 19]. The animals were then artificially ventilated with room air using a model 7025 Ugo Basile pump (56 strokes/min.; stroke volume: 20 ml/kg), as previously established [20]. After cervical bilateral vagotomy, catheters were placed in: (i) the right femoral vein, for the administration of antagonists, isometheptene racemate, (*R*)-isometheptene or (*S*)-isometheptene; and (ii) the left carotid artery, connected to a Grass pressure transducer (P23XL), for the recording of arterial blood pressure. Heart rate was measured with a tachograph (7P4F, Grass Instrument Co., Quincy, MA, USA) triggered from the blood pressure signal. Both heart rate and blood pressure were recorded simultaneously by a model 7D Grass polygraph (Grass Instrument Co., Quincy, MA, USA). The body temperature of each pithed rat was maintained at 37 °C by a lamp and monitored with a rectal thermometer.

### Experimental protocols

After a stable hemodynamic condition during at least 30 min, baseline values of heart rate and diastolic blood pressure (a more accurate indicator of peripheral vascular resistance than mean blood pressure) were determined. Subsequently, the 74 rats were randomly assigned into three main sets (*n* = 24, 40 and 10) for performing the following dose–response protocols. We a priori decided to investigate the pharmacological characteristics of the responses to the most potent enantiomer in more detail, since this enantiomer would most likely be responsible for the side-effects induced by isometheptene. In addition, we planned to study for the least potent enantiomer to which extent the cardiovascular responses were mediated by a direct or indirect, tyramine-like, mechanism of action.

### Protocol I. Comparative analysis of the effects produced by the isometheptene enantiomers

The first set of animals (*n* = 24) was divided into four groups (*n* = 6 each) that received consecutive i.v. bolus injections (0.03, 0.1, 0.3, 1 and 3 mg/kg) of, respectively: (i) isometheptene racemate; (ii) (*S*)-isometheptene; (iii) (*R*)-isometheptene; and (iv) equivalent volumes of physiological saline (1 ml/kg given 5 consecutive times; vehicle for dissolving isometheptene). The peak effects produced by each dose of the above compounds on heart rate and diastolic blood pressure were measured and compared, as previously reported [3]. The dose-intervals between the different doses of isometheptene (racemate and enantiomers) ranged between 5 and 15 min, as in each case we waited with the administration of the next dose until heart rate and diastolic blood pressure had returned to baseline values.

### Protocol II. Analysis of the pharmacological profile of the more potent enantiomer

The second set of animals (*n* = 40) was divided into three subsets (*n* = 25, 10 and 5)*.* The first subset (untreated; *n* = 25) received consecutive i.v. bolus injections of the more potent isometheptene enantiomer (0.03, 0.1, 0.3, 1 and 3 mg/kg). Then, this subset was divided into five groups (*n* = 5 each) that received i.v. bolus injections of, respectively: (i) physiological saline (1 ml/kg; vehicle for dissolving the antagonists); (ii) prazosin (0.1 mg/kg; α_1_-adrenoceptor antagonist); (iii) rauwolscine (0.3 mg/kg; α_2_-adrenoceptor antagonist); (iv) the combination of prazosin (0.1 mg/kg) plus rauwolscine (0.3 mg/kg); and (v) propranolol (1 mg/kg; nonselective β-adrenoceptor antagonist). The doses of the antagonists used were sufficient to completely block their corresponding receptors, as previously reported [3]. Subsequently, the responses to the above doses of the isometheptene enantiomer were elicited again 15 min after administration of the aforementioned compounds.

The second subset (reserpinized; *n* = 10) was pretreated with reserpine (5 mg/kg, i.p.) 24 h before the start of the experiments, as previously reported by our group [3], in order to eliminate the potential tyramine-like action exerted by the more potent isometheptene enantiomer. Then, this subset received sequential i.v. bolus injections of the isometheptene enantiomer (0.03-3 mg/kg) and was subsequently divided into two groups (*n* = 5 each) that were given i.v. bolus injections of, respectively: (i) physiological saline (1 ml/kg); and (ii) prazosin (0.1 mg/kg). After 15 min, the responses to the above isometheptene enantiomer doses were elicited again.

The third subset (sham; *n* = 5) was pretreated with the vehicle of reserpine (1 ml/kg of 5% ascorbic acid; i.p.) 24 h before the start of the experiments. Then, this subset received i.v. bolus injections of the isometheptene enantiomer as previously described.

### Protocol III. Effects of reserpine pretreatment on the effects of the less potent enantiomer

Finally, the third set of animals (*n* = 10) was divided into two groups (*n* = 5 each) that were pretreated i.p. (24 h before the start of the experiments) with, respectively: (i) 1 ml/kg of 5% ascorbic acid; and (ii) 5 mg/kg reserpine. Then, both groups received sequential i.v. bolus injections of the less potent isometheptene enantiomer (0.03-3 mg/kg).

### Data presentation and statistical evaluation

The number of animals was justified by statistical power calculation based on our previous studies with similar methodology [3]. All data in the text and figures are presented as mean ± S.E.M. The peak changes in the baseline values of heart rate and diastolic blood pressure elicited by the administration of isometheptene (racemate and enantiomers) were determined. The difference between the tachycardic and vasopressor responses to isometheptene within one subset/group of animals as well as between two groups (i.e. the more potent isometheptene enantiomer in reserpine- and vehicle-pretreated animals) were evaluated with the Student-Newman-Keuls’ test, once a two-way repeated measures analysis of variance had revealed that the samples represented different populations. Statistical significance was accepted at *P* < 0.05.

### Drugs

Apart from the anaesthetic (sodium pentobarbital), the drugs used in the present study were: isometheptene racemate (Carnick Laboratories, Cedar Knolls, NJ, USA); (*R*)-isometheptene and (*S*)-isometheptene (Tonix Pharmaceuticals Inc., New York, NY, USA); prazosin hydrochloride, rauwolscine hydrochloride, propranolol hydrochloride and reserpine (Sigma Chemical Co., St. Louis, MO, USA) All compounds were dissolved in physiological saline with the exception of reserpine, which was dissolved in 5% (w/v) ascorbic acid. A short period of heating was needed to dissolve prazosin. These vehicles had no significant effects on baseline heart rate or diastolic blood pressure, as previously reported by our group [3]. The doses mentioned in the text refer to the free base of substances in all cases.

## Results

### Systemic hemodynamic variables

The baseline values of heart rate and diastolic blood pressure in the pithed rats without reserpine were 260 ± 6 beats/min and 64 ± 1 mmHg, respectively. These variables did not significantly differ (*P* > 0.05) from those obtained in the animals pretreated with reserpine, i.e. 269 ± 4 beats/min and 67 ± 2 mmHg. Moreover, these hemodynamic variables remained without significant changes (*P* > 0.05) after administration of the i.v. bolus injections of saline, prazosin, rauwolscine, the combination prazosin plus rauwolscine or propranolol (not shown), as previously reported [3].

### Effects of isometheptene enantiomers on heart rate and diastolic blood pressure

**Fig. 1:**
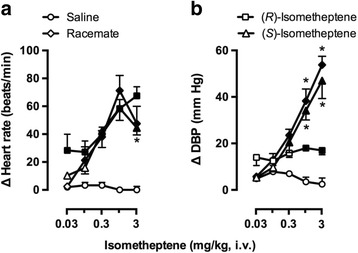
Effects of i.v. bolus injections of isometheptene enantiomers (0.03-3 mg/kg) or saline (1 ml/kg, given 5 times) on (**a**) heart rate; and (**b**) diastolic blood pressure (DBP) in pithed rats. Full symbols represent significant differences (*P* < 0.05) vs saline (vehicle). *P < 0.05 vs. the corresponding dose to (*R)*-isometheptene; n = 6 for each group

Figure 1 shows the effects produced by i.v. administration of isometheptene racemate, (*R*)*-*isometheptene and (*S*)*-*isometheptene on heart rate and diastolic blood pressure. These compounds produced: (i) dose-dependent tachycardic responses (left panel), with 3 mg/kg of these compounds representing the effects of a supramaximal dose; and (ii) dose-dependent vasopressor responses in the case of isometheptene racemate and (*S*)-isometheptene, while only smaller vasopressor responses (not dose-dependent; *P* < 0.05 vs. the corresponding dose of saline) were produced by the last two doses of (*R*)-isometheptene.

### Effect of antagonists on (*S*)-isometheptene-induced tachycardic and vasopressor responses

**Fig. 2:**
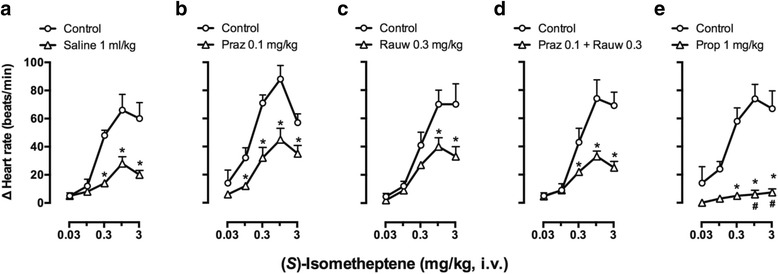
Effects of i.v. bolus injections of: (**a**) saline; (**b**) prazosin (Praz); (**c**) rauwolscine (Rauw); (**d**) the combination of Praz plus Rauw; or (**e**) propranolol (Prop) on the increases in heart rate (∆ Heart rate) produced by (*S*)-isometheptene (0.03-3 mg/kg, i.v.) in pithed rats. *P < 0.05 vs. the corresponding dose in the control curve; ^**#**^ *P* < 0.05 vs. the corresponding dose in the curve obtained after saline; *n* = 5 for each group

Figure 2 illustrates the tachycardic responses produced by (*S*)-isometheptene before and after different treatments. Indeed, these responses appeared to be tachyphylactic as they were significantly attenuated (*P* < 0.05) in control animals when repeating the dose-response curve after 1 ml/kg physiological saline (Fig. 2a). Furthermore, as compared to the tachycardic responses to (*S*)-isometheptene produced after saline, these responses were practically abolished after 1 mg/kg propranolol (Fig. 2e), but remained unaffected after administration of the other compounds (Figs. 2b, c, d).

**Fig. 3:**
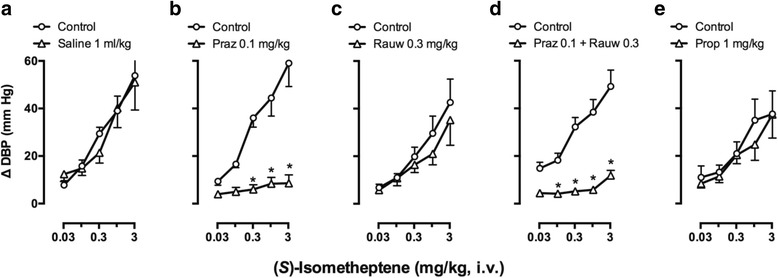
Effects of i.v. bolus injections of: (**a**) saline; (**b**) prazosin (Praz); (**c**) rauwolscine (Rauw); (**d**) the combination of Praz plus Rauw; or (**e**) propranolol (Prop) on the increases in diastolic blood pressure (∆ DBP) produced by (*S*)-isometheptene (0.03-3 mg/kg, i.v.) in pithed rats. *P < 0.05 vs. the corresponding dose in the control curve; *n* = 5 for each group

In contrast, as shown in Fig. 3, the vasopressor responses to (*S*)-isometheptene were: (i) reproducible as they remained practically unchanged after 1 ml/kg saline (Fig. 3a); (ii) similarly and markedly blocked (*P* < 0.05) after 0.1 mg/kg prazosin (Fig. 3b) or the combination of 0.1 mg/kg prazosin plus 0.3 mg/kg rauwolscine (Fig. 3d); and (ii) unaffected (*P* > 0.05) after 0.3 mg/kg rauwolscine (Fig. 3c) or 1 mg/kg propranolol (Fig. 3e). It is noteworthy that the blockade produced by the combination of prazosin plus rauwolscine did not significantly differ (*P* > 0.05) from that produced by prazosin alone.

### Cardiovascular effects of (*S*)-isometheptene in rats pretreated i.p. with reserpine or its vehicle

**Fig. 4:**
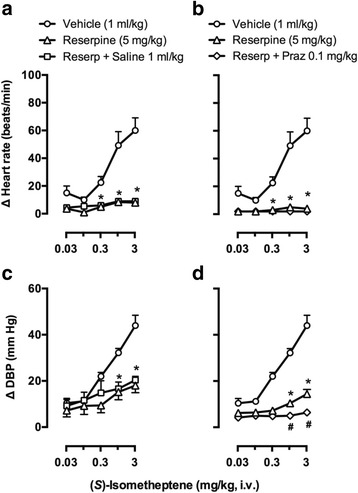
Effects of i.p. pretreatment with vehicle (5% ascorbic acid), reserpine (Reserp), reserpine followed by i.v. administration of saline or reserpine followed by i.v. administration of prazosin (Praz) on the increases in (**a**, **b**) heart rate (∆ Heart rate; upper panel) and (**c**, **d**) diastolic blood pressure (∆ DBP; lower panel) produced by (*S*)-isometheptene (0.03-3 mg/kg, i.v.) in pithed rats. *P < 0.05 vs. the corresponding dose in the vehicle curve; ^**#**^ *P* < 0.05 vs. the corresponding dose in the reserpine curve; *n* = 5 for each group

Figure 4 shows the effects of i.p. pretreatment with vehicle (5% ascorbic acid; 1 ml/kg), reserpine (5 mg/kg), reserpine followed by i.v. saline (1 ml/kg) or reserpine followed by i.v. prazosin (Praz; 0.1 mg/kg) on the increases in heart rate and diastolic blood pressure produced by (*S*)-isometheptene. Hence, in the vehicle-pretreated animals, (*S*)-isometheptene produced dose-dependent increases in heart rate (Figs. 4a, b) and diastolic blood pressure (Figs. 4c,d) as described above. In contrast, in the reserpine-pretreated animals: (i) (*S*)-isometheptene-induced tachycardic responses were practically abolished (as compared to the respective responses in vehicle-pretreated animals; *P* < 0.05), and the subsequent administration of saline (Fig. 4a) or prazosin (Fig. 4b) produced no further effect; and (ii) (*S*)-isometheptene-induced vasopressor responses were significantly attenuated (*P* < 0.05, but not abolished) and the subsequent administration of saline produced no further effect (Fig. 4c), but the subsequent administration of prazosin abolished these responses (*P* < 0.05; Fig. 4d).

### Cardiovascular effects of (*R*)-isometheptene in rats pretreated i.p. with reserpine or its vehicle

**Fig. 5:**
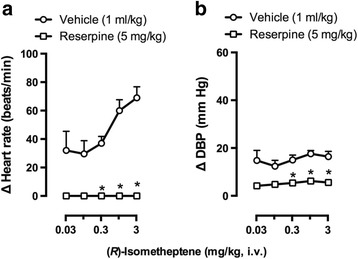
Effects of i.p. pretreatment with vehicle (5% ascorbic acid) or reserpine on the increases in: (**a**) heart rate (∆ Heart rate); and (**b**) diastolic blood pressure (∆ DBP) produced by (*R*)-isometheptene (0.03-3 mg/kg, i.v.) in pithed rats. *P < 0.05 vs. the corresponding dose in the vehicle curve; *n* = 5 for each group

As shown in Fig. 5, in the animals pretreated with vehicle (5% w/v ascorbic acid, 1 ml/kg; i.p.), (*R*)-isometheptene induced dose-dependent tachycardic responses (Fig. 5a) and small vasopressor responses (not dose-dependent; Fig. 5b). In contrast, in the animals pretreated with reserpine (5 mg/kg; i.p.), these tachycardic and vasopressor responses were practically abolished (*P* < 0.05 when compared to those produced by the corresponding dose in the vehicle curve).

## Discussion

### General

Apart from the implications discussed below, our study demonstrates the importance of investigating separately the enantiomers of a given compound. Indeed, the (*R*)- and (*S*)-enantiomers of a racemate may behave pharmacologically different from each other [21]. Thus, it is appropriate to consider each enantiomer as a separate pharmacological entity with different properties, unless proven otherwise [21]. In our study, the enantiomers of isometheptene displayed clear pharmacodynamic differences in their cardiovascular effects, and these differences could lead to the future usage of these enantiomers in different conditions.

### Systemic hemodynamic effects produced by the different treatments in pithed rats

The fact that the baseline values of diastolic blood pressure and heart rate remained without significant changes after prazosin, rauwolscine, the combination prazosin plus rauwolscine or propranolol (not shown), as previously reported [3], may be due to the fact that pithed rats are devoid of central and peripheral nervous influences. On this basis, it is most likely that the effect of any of these antagonists on the tachycardic and vasopressor responses to the isometheptene enantiomers involves a direct interaction with their corresponding receptors.

### Differential cardiovascular effects of the isometheptene enantiomers

In agreement with the sympathomimetic properties of isometheptene racemate in pithed rats [3], both enantiomers and the racemate produced dose-dependent increases in heart rate, although the tachycardic response produced by 3 mg/kg was not strictly dose-dependent (Fig. 1a) and apparently tachyphylactic. This finding implies a tyramine-like action (see below), as previously reported [3], and that such a high dose was supramaximal.

In contrast, (*R*)-isometheptene produced only small vasopressor responses at 1 and 3 mg/kg (which were not dose-dependent), while (*S*)-isometheptene and isometheptene racemate produced dose-dependent (and equipotent) vasopressor responses (Fig. 1b). The simplest interpretation of these findings indicates: (i) a differential pharmacological profile between (*R*)-isometheptene and (*S*)-isometheptene; and (ii) that the vasopressor effects of isometheptene racemate are mostly mediated by the (*S*)-enantiomer (see below). Since, overall, (*S*)-isometheptene displayed a higher potency to produce cardiovascular responses, we proceeded to analyse, in the first instance, the pharmacological profile of the receptors/mechanisms involved in the responses to (*S*)-isometheptene.

### The role of a tyramine-like action producing indirect stimulation of cardiac β-adrenoceptors in the tachycardic responses to (*S*)-isometheptene

The tachycardic responses to isometheptene racemate in pithed rats are mediated by an indirect tyramine-like action involving stimulation of propranolol-sensitive β-adrenoceptors, most likely of the β_1_-subtype [3]. In keeping with this finding, the tachycardic responses produced by (*S*)-isometheptene: (i) were highly tachyphylactic as they were not reproducible when repeating a second dose–response after saline (Fig. 2a); (ii) are mainly mediated by activation of propranolol sensitive β-adrenoceptors (Fig. 2e); and (iii) do not involve activation of prazosin-sensitive α_1_-adrenoceptors and/or rauwolscine-sensitive α_2_-adrenoceptors (Figs. 2b,c,d). Most notably, the fact that these responses were practically abolished after reserpine (Fig. 4a) reinforces the role of an indirect tyramine-like action in (*S*)-isometheptene-induced tachycardic responses (i.e. by displacing noradrenaline from sympathetic nerves which, in turn, would subsequently stimulate cardiac β-adrenoceptors). It is noteworthy that this dose-schedule with reserpine (i.e. 5 mg/kg, i.p.; −24 h) has been shown to abolish in pithed rats the tachycardic and vasopressor responses to the typical indirect sympathomimetic agent, tyramine [3].

### The role of both an indirect tyramine-like action and a direct stimulation of α_1_-adrenoceptors in the vasopressor responses to (*S*)-isometheptene

Unlike its tachycardic responses (see above), the vasopressor responses to (*S*)-isometheptene: (i) were reproducible as they remained without significant changes after saline (Fig. 3a); and (ii) are mainly mediated by α_1_-adrenoceptors as these responses were markedly blocked after prazosin (Fig. 3b), but not (*P* > 0.05) after rauwolscine (Fig. 3c) or propranolol (Fig. 3e) in doses high enough to completely block their respective receptors mediating cardiovascular responses [3]. Consistent with this suggestion, the combination prazosin plus rauwolscine produced a blockade (Fig. 3d) that did not significantly differ (*P* > 0.05) from that produced by prazosin alone (Fig. 3b). Furthermore, the fact that reserpine markedly attenuated (but did not abolish) these vasopressor responses (Fig. 4c), and that the subsequent administration of prazosin practically abolished these responses (Fig. 4d) suggests the involvement of a mixed effect of (*S*)-isometheptene, namely: (i) a major indirect (tyramine-like) mechanism; and (ii) a minor direct sympathomimetic mechanism mediated by stimulation of α_1_-adrenoceptors. In keeping with these findings, and as implied by other studies at the neuro-vascular junction [3, 22, 23]: (i) neuronally-displaced noradrenaline (i.e. by a tyramine-like action) primarily stimulates intrasynaptic α_1_-adrenoceptors; (ii) i.v. bolus of exogenous noradrenaline mainly activates extrasynaptic α_2_-adrenoceptors; and (iii) systemic vascular resistance (represented by diastolic blood pressure) is mainly modulated by α_1_-adrenoceptors.

Interestingly, the fact that (*R*)-isometheptene-induced tachycardic (Fig. 5a) and (weak) vasopressor (Fig. 5b) responses were abolished by reserpine suggests the exclusive role of a tyramine-like action in both responses (with no role of direct sympathomimetic actions). This implies that (*R*)-isometheptene would produce stoichiometric displacement of noradrenaline from the sympathetic neurons innervating the heart and resistance blood vessels, resulting in tachycardic and vasopressor responses mediated by stimulation of cardiac β- and vascular α_1_-adrenoceptors [3, 22, 23].

### Structure of the enantiomers and stereo-specificity of the α_1_-adrenoceptor

The different effects of the isometheptene enantiomers on the cardiovascular system in this study could also be explained by the chemical structures of these compounds. Indeed, there are some similarities between the molecular structures of isometheptene and noradrenaline, which may confer the capability of being recognized by the neuronal transporters, introduced into the synaptic vesicles and subsequently displace the stored catecholamines that, in turn, would interact with post-junctional adrenoceptors. The presence of an amine side chain in isometheptene provides the capability to stimulate directly the α_1_-adrenoceptor [23]. However, the presence of a chiral centre in the β carbon of the amine group could explain the differences in the pharmacological profile of both enantiomers, suggesting that (*R*)-isometheptene cannot interact with the α_1_-adrenoceptor, probably due to a steric impediment.

### Study limitations

The pithed rat model is a useful preparation for investigating the cardiovascular (side) effects of new developed antimigraine drugs (i.e. isometheptene enantiomers) and their mechanism of action. Since migraine-specific agents, namely triptans, are contraindicated for acute migraine attacks in patients with cardiovascular risk factors, new antimigraine drugs should ideally have a beneficial cardiovascular safety profile. The pithed rat model is appropriate to assess peripheral cardiovascular effects, however, it should be kept in mind that it excludes central nervous system mechanisms, which might be relevant in the clinical situation.

As migraine has a 2- to 3-fold higher prevalence in women, another limitation of our study is that we used only male rats. We deliberately chose to study male rats in this case since we wanted to limit experimental variation avoiding the effects of female hormones on vascular responsiveness, as it is known that noradrenaline vasopressor responses are altered in the presence of 17β-estradiol in this model [24]. Thus, future studies may focus on the antimigraine effects of the isometheptene enantiomers in female models during different hormonal cycle stages.

## Conclusions

Our findings show that both isometheptene enantiomers (*R* and *S*) are equipotent in producing tachycardic responses, and that these responses are mediated by a tyramine-like action (abolished by reserpine). Moreover, (*S*)-isometheptene produced greater dose-dependent vasopressor responses, while those produced by (*R*)-isometheptene were not dose-dependent. These effects are probably due to differences in their mechanism of action, namely: (i) a mixed sympathomimetic action for (*S*)-isometheptene (a tyramine-like action and a direct stimulation of α_1_-adrenoceptors); and (ii) exclusively a tyramine-like action for (*R*)-isometheptene.

Therefore, (*R*)-isometheptene may be responsible for the therapeutic action of isometheptene racemate and (*S*)-isometheptene (producing greater vasopressor responses) might be associated with the vasospasm described with the racemate. Accordingly, (*R*)-isometheptene may represent a superior therapeutic benefit as an antimigraine agent. Undoubtedly, further studies with (*R*)-isometheptene in human preparations and in clinical studies will shed further light on the potential role of imidazoline receptors in the pathophysiology of migraine.

## Abbreviations

i.p.: Intraperitoneal
i.v.: Intravenous
MMA: Middle meningeal artery
